# Survival Status and Its Determinants among Under-Five Children with Severe Acute Malnutrition Admitted to Inpatient Therapeutic Feeding Centers in South Wollo Zone, Amhara Region, Ethiopia

**DOI:** 10.1155/2019/2643531

**Published:** 2019-03-31

**Authors:** Seid Legesse Hassen, Ayalew Astatkie, Tefera Chanie Mekonnen, Getahun Gebre Bogale

**Affiliations:** ^1^Research and Technology Transformation Directorate, Amhara Public Health Institute, Dessie Branch, Dessie, Ethiopia; ^2^School of Public Health, College of Medicine and Health Sciences, Hawassa University, Hawassa, Ethiopia; ^3^Department of Public Health, College of Medicine and Health Sciences, Wollo University, Dessie, Ethiopia; ^4^Department of Basic Health Science, Dessie College of Health Science, Dessie, Ethiopia

## Abstract

**Background:**

Under nutrition is one of the leading causes of morbidity and mortality in under-five children in developing countries including Ethiopia. In Ethiopia, many children with severe acute malnutrition (SAM) are treated at inpatient therapeutic feeding centers. However, the survival status and its determinants are not well understood. Therefore, the aim of this study was to estimate the survival status and its determinants among under-five children with severe acute malnutrition admitted to inpatient therapeutic feeding centers (ITFCs).

**Methods:**

A record review was conducted on 414 under-five children who were admitted with severe acute malnutrition to ITFCs in South Wollo Zone, northeast Ethiopia, between September 11, 2014, and January 9, 2016. Data were entered into Epi-Info version 7.2 and analyzed using SPSS version 20. Life table analysis was used to estimate cumulative proportion of survival. The relationship between time to recovery and covariates was determined using Cox-proportional hazards regression model. *p* < 0.05 was used to declare presence of significant association between recovery time and covariates.

**Results:**

Of the total children recorded, 75.4% of children were recovered and discharged, 10.3% were defaulters, 3.4% died, 7.4% were nonresponders, and 3.4% were unknown. The mean (±standard deviation) time to recovery was 12 (±5.26) days, whereas the median time to recovery was 11 (interquartile range of 8–15) days. Children's breastfeeding status at admission (AHR: 1.42, 95% CI: 1.10, 1.83) and children without comorbidities at admission (AHR: 1.44, 95% CI: 1.03, 2.00) had statistically significant effect on time to recovery from SAM.

**Conclusion:**

All treatment responses in this study were within the recommended and acceptable range of global standards. Policy makers, health facilities, and care providers may need to focus on the importance of breastfeeding especially for those under two years of age and give emphasis for cases with comorbidities.

## 1. Introduction

Globally, under nutrition remains one of the most common causes of morbidity and mortality among children. Nearly 20 million children below 5 years of age suffer from wasting and are at risk of death or severe impairment of growth and psychological development [1]. Of these, over 90% are found in South and Southeast Asia and sub-Saharan Africa. About one million deaths occur annually among children under five years of age in developing countries [2]. Ethiopia has one of the highest child mortality rates in the world with 57% of all deaths in children who have stunting and wasting as the underlying cause [3]. The percentages of children with stunting, wasting, and underweight at national level were 40, 9, and 26, respectively [4]. The minimum international standard set for management of severe acute malnutrition (SAM) is a recovery rate of at least 75% and death rate less than 10% [5, 6].

Studies conducted in Ethiopia revealed that the recovery rates and median survival times of under-five children with SAM were ranged from 83% to 89% and 14 days to 25 days, respectively [7–11]. Previous research studies conducted in the country identified determinants of children's survival. Sex of child, medical comorbidities, treatment and follow-up status, and clinical diagnosis of SAM were associated to survival of children [12–16]. The Ethiopian Ministry of Health established inpatient therapeutic feeding centers in the health facilities to reduce SAM-related mortalities of under-five children. Despite the existence of SAM management at hospital or health center level in every corner of the country, deaths due to SAM is indicated to be still high, and little is known about the recovery time and its determinants from SAM particularly in under-five children admitted to inpatient therapeutic feeding centers (ITFCs) [17].

This study aimed to estimate the survival status and its determinants among under-five children with severe acute malnutrition admitted to inpatient therapeutic feeding centers (ITFCs) in South Wollo Zone, northeast Ethiopia, over the past two and half years. Thus, these findings would be essential to provide evidences for decision makers, health facilities, and care providers to take measures to target those children at highest risk of slow recovery.

## 2. Methods

### 2.1. Study Design, Period, and Setting

A facility-based retrospective record review was employed to estimate survival status and its determinants among under-five children with severe acute malnutrition admitted to ITFCs in South Wollo Zone, northeast Ethiopia, from September 11, 2014, to January 9, 2016. The study was conducted in South Wollo Zone, which is bordered on the south by North Shewa, on the west by East Gojjam, on the northwest by South Gondar, on the north by North Wollo, on the northeast by Afar Region, and on the east by the Oromia Zone and Argobba special district. It is one of the 11 Zones found in Amhara National Regional State. The zone has twenty-two districts. The zone has a total population of 2,518,862, of whom 1,248,698 are men and 1,270,164 women. It has a total area of 17,067.45 square kilometers. The population density of the zone is 147.58 per square kilometer. About 12% of the population inhabits urban areas. Children under five years of age constitute 352,642 of the population [18]. According to the South Wollo Zone Health Department report, the zone has seven governmental hospitals, one hundred thirty-five health centers, and four hundred ninety-six health posts. Most of these people are seasonal agriculturalists and prone to recurrent drought and food insecurity. The common health problems in this zone are diarrhea, under-five pneumonia, other communicable diseases, and malnutrition [19].

### 2.2. Study Participants

Under-five children in the study area were screened for signs of SAM and diagnosed based on anthropometric measurements and examination of the feet for bilateral pitting edema. They were admitted to ITFCs after fulfilling the criteria of admission. They were admitted if the W/H was <70% of the median WHO growth reference, or if the mid upper arm circumference (MUAC) was less than 11 cm, or children with bilateral pedal edema [20]. On admission, malnourished patients were assessed for hydration status, anemia, and signs of infections. They were given oral dose of vitamin A, mebendazole, folic acid, and a course of amoxicillin for five days. Rehydration solution for malnutrition (ReSoMal) was used for treating dehydrated cases, and drugs such as gentamicin, chloramphenicol, or quinine were used based on causes of infections. Treatment of severe malnutrition was divided into three phases: the first phase (phase I), transition phase, and phase II. In phase I, health workers resuscitated patients, treated for infections, restored electrolyte balance, and prevented hypoglycemia and hypothermia on indication. F75 milk (formula 75 that contains 75 kcal in 100 ml, minerals, and proteins) was used during phase I treatment; malnourished cases who responded on treatment by return of appetite, beginning of loss of edema, and no intravenous line or nasogastric tubes were transferred to transition phase to receive F100 (formula 100 that contains 100 kcal in 100 ml). Afterwards, cases were transferred to phase II after they gained good appetite (finish 90% of F100 prescribed for transition phase) and cleared edema. The discharge criterion for children with SAM is W/H ≥ 85% [21].

### 2.3. Sample Size and Sampling Procedures

**Figure 1:**
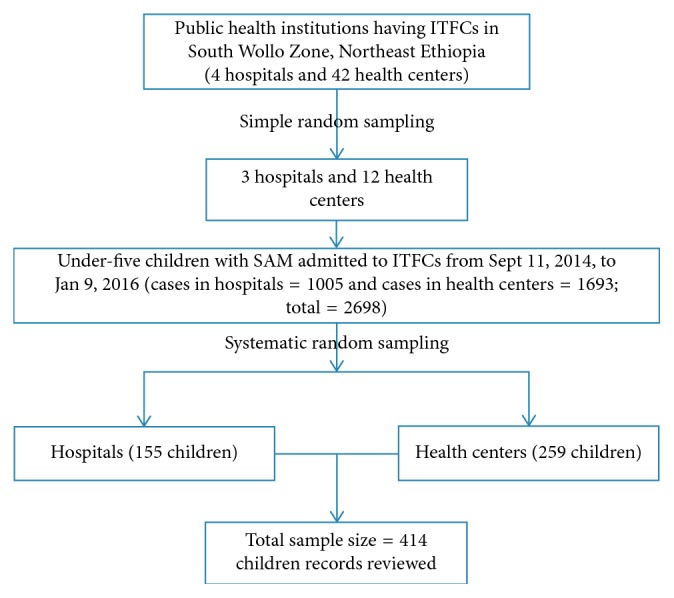
Schematic diagram of sampling procedures in South Wollo Zone, northeast Ethiopia, 2014–2016.

Sample size was determined based on the assumptions with recovery rate of 93.3%, hazard ratio of 0.38 [9], 5% margin of error, 95% CI, and power of 80%. Thus, considering after 10% adding for incomplete records, the total number of study sample size was 414. There are 46 public health facilities that have ITFCs in South Wollo Zone. Of these, 15 public health facilities were randomly selected and included in the study. After proportional allocation was employed for each health facility, a total sample size of 414 under-five children with SAM were selected for the study using systematic random sampling method (Figure 1).

### 2.4. Data Analysis

The data were collected by reviewing records from ITFCs' registration book and individual follow-up chart using standardized and pretested data collection tool. Data were entered into Epi-Info version 7.2 and analyzed using SPSS version 20. The outcome variable in this study was time to recovery (stabilized and discharge to outpatient therapeutic centers) following treatment for SAM. Children who defaulted from treatment, died, or became nonresponders were considered to be censored. Finally, the outcome of each subject was dichotomized into censored or survived/recovered.

For comparison, demographic characteristics were described in terms of mean (standard deviation) and median (interquartile range) for continuous data and frequency distribution for categorical data. Determinants were assessed by bivariate and multivariable screening models. Variables in the bivariate analysis of sociodemographic and admission information, medical comorbidities, treatment given, and follow-up with respect to survival status, which were found at *p* value <0.20, were further considered in to multivariable Cox-regression model. The enter method of selection for final model was used. Crude and adjusted hazard ratios with their 95% confidence interval (CI) were estimated, and *p* < 0.05 was used to declare presence of significant association between time to recovery and covariates. Life table analysis was used to estimate cumulative proportion of survival among children with SAM at different time points. Kaplan–Meier test was used to show nutritional survival time after initiation of inpatient treatment, and log rank survival curve was used to compare median survival time between groups.

## 3. Results

### 3.1. Sociodemographic and Admission Characteristics

Four hundred six children were included in the study with response rate of 98%. From these children records, 229 (56.4%) were male and 177 (43.6%) were female. Their mean (±SD) age was 16.7 (±10.96). Of the total study subjects admitted to ITFCs, 36 (8.9%) were under six months. Three hundred thirty (81.3%) children were admitted from rural residences. Of the total of 406 children records, 308 (75.9%) were clinically diagnosed as marasmus, 60 (14.8%) were kwashiorkor, and 38 (9.4%) were marasmic-kwashiorkor at admission. Three hundred seventy-three (65.4%) of the admission criteria was W/H <70%. Among edematous cases, forty nine (50%) of them had edema in both feet, in legs, and in hands or face (grade 3). Among the total children, 394 (97%) of them were newly admitted and the rest 12 (3%) were repeat. One hundred eighteen (29.1%) children were admitted during spring and 117 (28.8%) children were admitted during summer (rainy) seasons. However, the rest, 92 (22.7%) and 79 (19.5%), were admitted during autumn (crop harvesting time) and winter (sunny) seasons, respectively. When they were tested for appetite at admission, 235 (57.9%) failed and 171 (42.1%) passed. When they checked for breastfeeding status at admission, 286 (70.4%) had been breastfed and 120 (29.6%) had not been breastfed (Table 1).

### 3.2. Anthropometric Measurements

The mean (±SD) weight of children was 6.29 (±1.95) kilograms at admission and 6.75 (±1.99) kilograms at discharge. The children's mean (±SD) height was 69.76 (±10.04) centimeters at admission and 70.12 (±9.39) centimeters at discharge. The mean (±SD) mid upper arm circumference of children was 103.00 (±8.39) millimeters at admission and 112.82 (±9.48) millimeters at discharge.

### 3.3. Medical Comorbidities, Treatment Given, and Follow-Up

Of the total 406 children admitted to inpatient therapeutic feeding centers (ITFCs), 355 (87.4%) had medical comorbidities. Of all comorbidities, fever was 210 (51.7%), cough/pneumonia was 179 (44.1%), and diarrhea was 164 (40.4%). Among diarrheal cases, 113 (68.9%) of the children had watery diarrhea and 98 (9.8%) had no dehydration. Of the total 406 children admitted to ITFCs, 386 (95.1%) had been given antibiotics, 260 (64.0%) vitamin A, 180 (44.3%) deworming, and 174 (42.9%) zinc tablet. However, the rest did not take any medication. Almost all of the children, 387 (95.3%), were immunized. Of all children admitted, 263 (64.8%) had been given paracetamol tablet/syrup, 57 (14%) ReSoMal, and 201 (49.5%) special (like ceftriaxone, ringer lactate, normal saline) IV medication (Table 2).

### 3.4. Treatment Response and Length of Stay for Recovery

Of the total 406 children admitted to ITFCs, the recovery rate from SAM was 306 (75.4%), whereas the remaining 14 (3.4%) children died, 42 (10.3%) defaulted, 30 (7.4%) were nonresponders, and 14 (3.4%) were unknown.

The overall average (±SD) length of stay for the edematous children with SAM was 12.5 (±5.6) days, and children with SAM that stayed almost similar in the ITFCs had severe wasting (12.0 (±5.1)). The median (IQR) length of stay for children who were diagnosed with edematous and severely wasted was 12.5 (8, 16) and 11.0 (8, 15) days, respectively.

### 3.5. Determinants of Nutritional Recovery Time

After minimizing the confounders, only two variables were associated with time to recovery. Children who had breastfeeding status and children who had no comorbidities at admission had shown significant association with time to recovery from SAM. Children who had been breastfed at admission were 1.42 times (AHR: 1.42, 95% CI: 1.10, 1.83) more likely to have shorter recovery time as compared to the nonbreastfed at any time. Children with the absence of comorbidities at admission were 1.44 times (AHR: 1.44, 95% CI: 1.03, 2.00) more likely to have shorter recovery time than who had comorbidities at any time (Table 3).

### 3.6. Life Table and Kaplan–Meier Analysis

The life table showed that cumulative probability of staying in the program was 92% at third day, 72% at sixth day, 47% at ninth day, 30% at twelfth day, and zero percent at 33 days of admission (Table 4).

**Figure 2:**
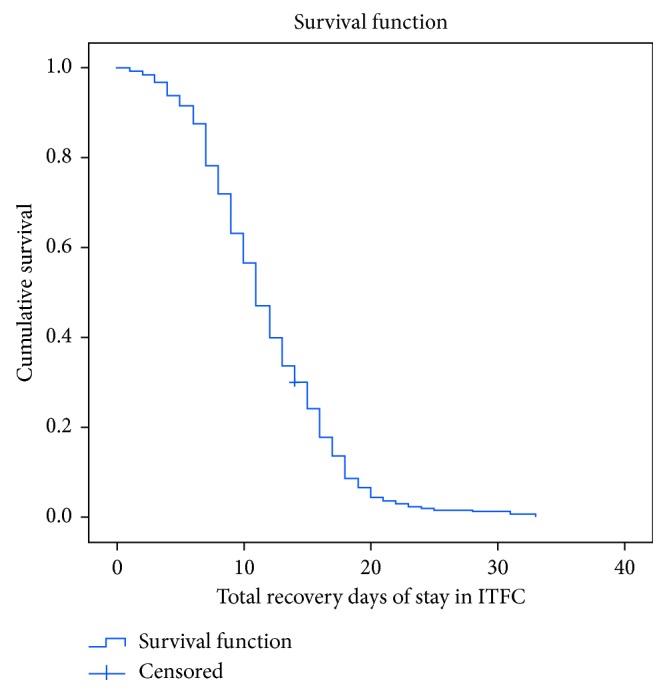
Overall Kaplan–Meier recovery estimate of under-five children with SAM treated under ITFCs in South Wollo Zone, Ethiopia, between September 11, 2014, and January 9, 2016.

The overall Kaplan–Meier curve (Figure 2) showed a step function and was used to estimate the survival function from lifetime data. Time to recovery of 286 children with breastfeeding and 120 children without breastfeeding was compared. The Kaplan–Meier (KM) survival curve for breastfeeding status at admission illustrated that the treatment recovery time of children with breastfeeding was better than that of children without breastfeeding at admission.

**Figure 3:**
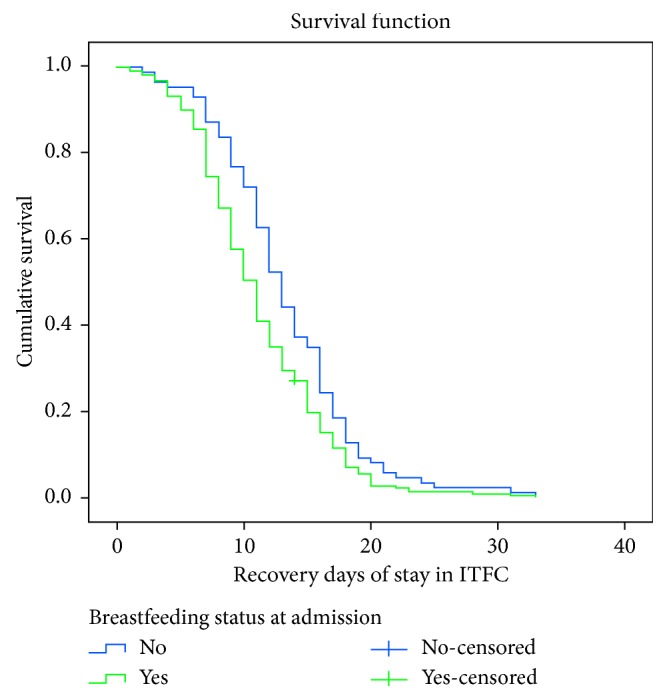
Kaplan–Meier recovery estimate for children who were breastfed and not breastfed at admission under inpatient therapeutic feeding centers in South Wollo Zone, northeast Ethiopia, between September 11, 2014, and January 9, 2016 (log rank (Mantel–Cox) *X*^2^ = 7.995, *p* value = 0.005).

The significance of the observed differences of the Kaplan–Meier survival curves (times) among the two groups of children was assessed using log rank test. As a result, breastfeeding status of children was found to have statistically significant association (*X*^2^=7.995, *p* < 0.005) (Figure 3).

**Figure 4:**
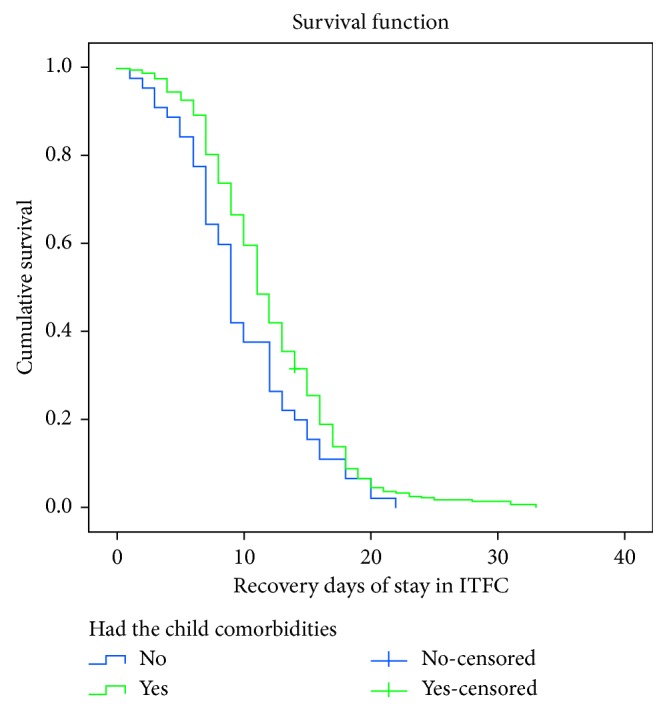
Kaplan–Meier recovery estimate for children with the absence of comorbidities and with comorbidities at admission under ITFCs in South Wollo Zone, northeast Ethiopia, between September 11, 2014, and January 9, 2016 (log rank (Mantel–Cox) *X*^2^ = 4.923, *p* value = 0.027).

The time to recovery of 355 children with medical complications and 55 children without complications at admission was compared. The KM survival curve for children without complications at admission illustrated that the treatment outcome of children without complications was better than that of children with comorbidities status at admission. The significance of the observed differences of the KM survival curves (times) among the two groups of children was assessed using log rank test. As a result, children with no comorbidities were found to have statistically significant association (*X*^2^=4.923, *p* < 0.0271) (Figure 4).

## 4. Discussion

Among 414 eligible study subjects, 406 under-five children were considered in this study and eight were missed due to incomplete data. More than 75% of them were recovered from SAM, while the remaining were censored. The survival rate was 75.4%. The mean time-to-cure was 12 (±5.26) days, whereas the median time-to-cure was 11 (IQR: 8–15) days.

The mean (±SD) age of children with SAM was 17 (±11) months, which was consistent with similar studies conducted in developing countries [9, 19, 22]. Majority (85%) of admissions in ITFCs were between 1–24 months of age due to higher risk factors for malnutrition [19]. In the current study, survival (recovery) rate was 75.4%, defaulter was 10.3%, and death rates were 3.4%. Ethiopia is currently using a protocol for the management of severe acute malnutrition that is an update of existing guideline. As mentioned in this protocol, the minimum acceptable reference values which have been developed by Sphere project are >75% recovery, <15% defaulter, and <10% death rates [23]. Thus, our finding was within the acceptable range of this protocol. This is also consistent to other studies in the country [8, 10]. This might be due to proper use of clinical management protocols which is capable of reducing case fatality rates [19]. In this study, the clinical classifications of SAM reported as overall proportion of recovery were 76.1% with marasmus, 14.7% with kwashiorkor, and 9.2% with marasmic-kwashiorkor. This finding is in line with other studies conducted in north Ethiopia [24], whereas it is dissimilar with other study conducted outside the country [11]. It also contrasted with the national report [4]. This might be due to differences in feeding culture and socioeconomic status. This study indicated that the mean time taken to recover from SAM for both children diagnosed with edematous (12.5 days) and severe wasting (12.03 days) was almost similar. It was found to be contrasting with other findings in the country in which those children who were diagnosed with marasmus at admission stayed longer before recovery than their kwashiorkor counterparts [10].

This study revealed that the overall average length of stay in South Wollo Zone in ITFCs was 12.27 days (12.5 days for edematous and 12.03 days for severe wasting children). It is consistent with the minimum international standard set for management of severe acute malnutrition which is an average length of stay less than 30 days [23]. The mean and median time-to-cure from SAM for patients in the zone were 12 (±5.26) days and 11 days (IQR: 8–15 days) with minimum and maximum cure time of 1 and 33 days, respectively. A program is well functioning and considered acceptable if the length of stay in a hospital is less than 28 days. This implies that the program was acceptable as per the national standard [19]. This was consistent with another study conducted in the country [7]. Majority of the children recovered from SAM at first week were 86 (28%), at second week 265 (58.5%), and at third week 299 (11%). This was in line with findings of Jimma University Specialized Hospital [25].

Of the total study subjects, severe wasting (marasmus) was the most common one (75.9%) which was similar with other studies [11, 14, 26]. Fever, pneumonia, and diarrhea were the most common comorbidities. This was almost similar with another study conducted in Zambia University Teaching Hospital [27].

In final analysis model (Cox regression), breastfeeding status and absence of medical complication at admission were found to be independent predictors of shorter recovery time. Other covariates did not have an impact. Children with breastfeeding at admission had shorter recovery rate compared to their counterparts at any time. In KM analysis, the two survival curves were significantly different. It implied that breastfeeding may always be the preferred option in most contexts to increase recovery rate of children from severe acute malnutrition. This was in line with the national severe acute malnutrition guidelines [19, 23]. Even advice on breastfeeding for infants is a recommended treatment plan for uncomplicated SAM and moderate acute malnutrition [28]. It should also be offered to malnourished children before the diet and always on demand [19]. Children with the absence of comorbidities at admission had shorter recovery rate than who have comorbidities at any time [9]. Comorbidities may prolong the duration of stay in the ITFCs and may increase death rate [26]. This finding was in agreement with other studies conducted in Ethiopia and other developing countries [11, 13, 14, 29].

Since the study was based on secondary data, incomplete registration was observed in some anthropometric measurements at discharge, and even measurement errors had been introduced. Among treatment responses, “unknown cases” were categorized as “censored”, which may compromise the children's recovery rate from SAM. Thus, it may affect the findings through under or over reporting. Further prospective study is recommended to overcome these threats.

## 5. Conclusions

Our findings showed that the overall survival/recovery rate, death rate, and time to recovery from SAM were as per the international and national minimum standards set for SAM management. Children with breastfeeding status at admission had a shorter time of recovery from SAM. Children with medical complications at admission had a longer time of recovery from SAM. Therefore, enhancing breastfeeding for children up to at least two years of age and early detection of under-five children's morbidities are largely important to improve time of recovery from severe acute malnutrition. Additionally, in the community, breastfeeding promotion should also prevent SAM, and treatment of nonbreastfeeding children with SAM should include breastfeeding support for mothers and relactation where possible.

## Figures and Tables

**Table 1:** Sociodemographic and admission characteristics of under-five children with SAM in ITFCs in South Wollo Zone, northeast Ethiopia, from September 11, 2014, to January 9, 2016.

Variables	Frequency	Percentage
Age of child in months (*n*=406)		
1–24 months	345	85.0
25–59 months	61	15.0

Sex (*n*=406)		
Male	229	56.4
Female	177	43.6

Place of residence (*n*=406)		
Urban	76	18.7
Rural	330	81.3

Clinical classification/diagnosis(*n*=406)		
Only edema (kwashiorkor)	60	14.8
Only wasting (Wt/Ht)	308	75.9
Both edema and wasting (marasmic-kwashiorkor)	38	9.4

Admission criteria (*n*=406)		
Wt/Ht < 70%	265	65.4
W/A < 60% (marasmus)	15	3.7
Edema (kwashiorkor)	71	17.4
MUAC <110 mm	55	13.5

Grades of nutrition edema (*n*=98)		
Grade 1 (+)	15	15.3
Grade 2 (++)	34	34.7
Grade 3 (+++)	49	50.0

Admission status (*n*=406)		
New	394	97.0
Repeat	12	3.0

Season of admission (*n*=406)		
Summer (Jun. to Aug.)	117	28.8
Autumn (Mar. to May)	92	22.7
Winter (Dec. to Feb.)	79	19.5
Spring (Sep. to Nov.)	118	29.1

Appetite test at admission (*n*=406)		
Pass	171	42.1
Fail	235	57.9

Breastfeeding status at admission (*n*=406)		
No	120	29.6
Yes	286	70.4

**Table 2:** Medical comorbidities, treatment given, and follow-up characteristics of under-five children with SAM in ITFCs in South Wollo Zone, northeast Ethiopia, from September 11, 2014, to January 9, 2016.

Variables	Frequency	Percentage
Had the child comorbidities (*n*=406)		
No	51	12.6
Yes	355	87.4

Which comorbidities?		
Fever (body temperature >37.5°C)	210	51.7
Diarrhea	164	40.4
Cough/pneumonia	179	44.1
Malaria	15	3.7
Vomiting	110	27.1
Sepsis	28	6.9
Superficial infection (skin or ear infection)	42	10.3
Severe anemia	19	4.7
HIV/AIDS	7	1.7
Hypothermia (body temperature ≤35°C)	11	2.7
TB	4	1.0
Others	6	1.5

Type of diarrhea (*n*=164)		
Watery	113	68.9
Dysentery	51	31.1
Degree of dehydration (*n*=164)		
No dehydration	98	59.8
Some dehydration	61	37.2
Severe dehydration	5	3.0

Was the child given antibiotic? (*n*=406)		
No	20	4.9
Yes	386	95.1

Was the child given vitamin A? (*n*=406)		
No	146	36.0
Yes	260	64.0

Was the child given deworming? (*n*=406)		
No	226	57.7
Yes	180	44.3

Was the child given zinc tab? (*n*=406)		
No	232	57.1
Yes	174	42.9

Immunization status (*n*=406)		
Fully immunized	294	72.4
Up to date	93	22.9
Not yet immunized	19	4.7

Was the child given paracetamol tab/syrup (*n*=406)		
No	143	35.2
Yes	263	64.8

Was the child given ReSoMal (*n*=406)		
No	349	86.0
Yes	57	14.0

Was the child given special IV medication? (*n*=406)		
No	205	50.5
Yes	201	49.5

**Table 3:** Determinants of time to recovery for under-five children with SAM in ITFCs in South Wollo Zone, northeast Ethiopia, between September 11, 2014, and January 9, 2016.

Variables	Survival status		Crude hazard ratio (95% CI)	*p* value	Adjusted hazard ratio (95% CI)	*p* value
	Recovered	Censored				
Age (*n*=406)						
1–24 months	259	86	1.19 (0.87, 1.62)	0.276		
25–59 months	47	14	1			

Sex (*n*=406)						
Male	198	31	1			
Female	108	69	1.08 (0.85, 1.36)	0.549		

Place of residence (*n*=406)						
Urban	65	11	1.12 (0.85, 1.47)	0.432		
Rural	241	89	1			

Clinical diagnosis (*n*=406)						
Kwashiorkor	45	15	1		1	
Marasmus	233	75	1.26 (0.91, 1.75)	0.161	1.19 (0.86, 1.66)	0.294
Both Mar-Kwas	28	10	1.34 (0.83, 2.17)	0.228	1.34 (0.83, 2.16)	0.238

Admission status (*n*=406)						
New	296	98	1			
Repeat	10	2	0.77 (0.41, 1.45)	0.414		

Season of admission (*n*=406)						
Summer	106	11	1.02 (0.75, 1.38)	0.913		
Autumn	82	10	1.11 (0.80, 1.53)	0.545		
Winter	51	28	1.08 (0.75, 1.56)	0.676		
Spring	67	51	1			

Appetite test at admission (*n*=406)						
Pass	129	42	1.05 (0.93, 1.17)	0.439		
Fail	177	58	1			

Breastfeeding status at admission (*n*=406)						
No	87	33	1		1	
Yes	219	67	1.39 (1.08, 1.79)^*∗*^	0.010	1.42 (1.10, 1.83)^*∗*^	0.008

Had the child comorbidities (*n*=406)						
No	45	6	1.18 (1.01, 1.38)^*∗*^	0.041	1.44 (1.03, 2.00)^*∗*^	0.031
Yes	261	94	1		1	

Type of diarrhea (*n*=406)						
Watery	79	34	0.87 (0.59, 1.29)	0.495		
Dysentery	37	14	1			

Degree of dehydration (*n*=406)						
No dehydration	73	25	1.11 (0.77, 1.56)	0.558		
Some dehydration	40	21	0.96 (0.66, 1.39)	0.813		
Severe de	3	2	1			

Antibiotic given (*n*=406)						
No	14	6	1			
Yes	292	94	0.86 (0.50, 1.47)	0.579		

Vitamin A given (*n*=406)						
No	106	40	1			
Yes	200	60	1.16 (0.91, 1.47)	0.223		

Deworming given (*n*=406)						
No	168	58	1			
Yes	138	42	0.99 (0.79, 1.25)			

Zinc tab given (*n*=406)						
No			1			
Yes			0.91 (0.72, 1.14)	0.403		

Immunization status (*n*=406)						
Fully immunized	224	70	1.08 (0.86, 1.34)	0.518	1.82 (0.99, 3.32)	0.053
Up to date	70	23	1.20 (0.94, 1.55)	0.151	1.86 (1.00, 3.50)	0.052
Not yet immunized	12	7	1		1	

Paracetamol tab/syrup given (*n*=406)						
No	108	35	1			
Yes	198	65	0.92 (0.72, 1.16)	0.463		
ReSoMal given (*n*=406)						
No	267	82	1			
Yes	39	48	0.98 (0.70, 1.37)	0.901		

IV medication given (*n*=406)						
No	156	49	0.94 (0.84, 1.06)	0.314		
Yes	150	51	1			

NB. ^*∗*^*p* value <0.05.

**Table 4:** Life table for under-five children with SAM in ITFCs in South Wollo Zone, northeast Ethiopia, between September 11, 2014, and January 9, 2016.

Interval start time (days)	Number entering interval	Number exposed to risk	Number of terminal events	Proportion terminating	Proportion surviving	Cumulative proportion surviving at end of interval
0	306	306	5	0.02	0.98	**0.98**
3	301	301	21	0.07	0.93	**0.92**
6	280	280	60	0.21	0.79	**0.72**
9	220	220	76	0.35	0.65	**0.47**
12	144	144	53	0.36	0.63	**0.30**
15	91	91	50	0.55	0.45	**0.14**
18	41	41	28	0.68	0.32	**0.04**
21	13	13	6	0.46	0.54	**0.02**
24	7	7	2	0.29	0.71	**0.02**
27	5	5	1	0.20	0.80	**0.01**
30	4	4	2	0.50	0.50	**0.01**
33	2	2	2	1.00	0.00	**0.00**

## Data Availability

The data used to produce this manuscript are available in Epi-Info version 7.2 and SPSS version 20 databases and the authors are prepared to share their data on request recognizing the benefits of such transparency.

## Abbreviations

AHR: Adjusted hazard ratio
CI: Confidence interval
IQR: Interquartile range
ITFCs: Inpatient therapeutic feeding centers
KM: Kaplan–Meier
MUAC: Mid upper arm circumference
ReSoMal: Rehydration solution for malnutrition
SAM: Severe acute malnutrition
SD: Standard deviation
W/H: Weight for height
WHO: World Health Organization.
